# Influence of Conserved and Hypervariable Genetic Markers on Genotyping Circulating Strains of *Neisseria gonorrhoeae*

**DOI:** 10.1371/journal.pone.0028259

**Published:** 2011-12-07

**Authors:** Sinisa Vidovic, Greg B. Horsman, Mingmin Liao, Jo-Anne R. Dillon

**Affiliations:** 1 Vaccine and Infectious Disease Organization, University of Saskatchewan, Saskatoon, Saskatchewan, Canada; 2 Saskatchewan Disease Control Laboratory, Regina, Saskatchewan, Canada; 3 Department of Biology, University of Saskatchewan, Saskatoon, Saskatchewan, Canada; St. Petersburg Pasteur Institute, Russian Federation

## Abstract

Presently there is no vaccine against *Neisseria gonorrhoeae* and therefore accurate information on gonococcal transmission plays a crucial role for interventions designed to limit the spread of infections caused by this microorganism. We evaluated the impact of two different categories of genetic markers, (i) concatenated sequences of 10 housekeeping genes and (ii) hypervariable *porB* DNA sequences, on the genetic relatedness and subsequently on genotyping analysis of this human pathogen. Eighty gonococcal isolates from Canada, China, the US, Argentina, Venezuela and Chile, collected over different times, were analyzed. Our results show that the choice of genetic marker had a profound effect on the interpretation of genotyping results associated with *N. gonorrhoeae*. The concatenated sequences of the housekeeping genes preserved the genetic relatedness of closely related isolates, enabling detection of the predominant strains circulating within a community (Saskatchewan, Canada) over an extended period of time. In contrast, a genetic marker based on antigen gene, *porB*, may lead to a failure to detect these predominant circulating strains. Based on the analysis of the DNA sequences of the 10 housekeeping genes, we identified two major clonal complexes, CC33 and CC22, which comprised STs from China, and Argentina as well as two STs from Canada. Several minor clonal complexes were observed among isolates from Saskatchewan. eBURST analysis suggested that the majority of the tested gonococcal isolates from Saskatchewan, Canada were endemic, with only a couple of genotypes introduced.

## Introduction

*Neisseria gonorrhoeae*, a fastidious Gram-negative diplococcus, is an obligate human pathogen infecting mucosal surfaces and causes the gonorrhoea, the most predominant bacterial sexually transmitted infection worldwide. The spread of the organism occurs through direct person to person contact and infections occur exclusively in humans. In males, infections commonly cause urethritis and cervicitis in females. Rectal and pharyngeal infections can occur in both sexes and complications from adjacent spread of the microorganisms along mucosal surfaces may result in endometritis, salpingitis, and peritonitis in women, and bartholinitis in men. Both sexes may risk infertility if the infection is left untreated and the organism is one of the most common causes of female infertility worldwide [1]. Asymptomatic infections, especially common in women [2], play an important role in the dissemination of the infection and its persistence in humans. Presently there is no vaccine against *N. gonorrhoeae* and therefore preventive measures based on clinical and epidemiological analyses the identification of the organism and effective antimicrobial treatment are key strategies used to limit the spread of the infection. Thus, a desirable method for typing *N. gonorrhoeae* isolates so that isolate relatedness can be ascertained over long and short time periods should possess a high resolution power, reproducibility, comparability and objectivity.

A number of typing methods for *N. gonorrhoeae* have been developed over the years, ranging from auxotyping [3], [4], serotyping [5] and plasmid typing [6] to various DNA based methods including older methods which focus on the analysis of band patterns e. g. opa typing, randomly amplified polymorphic DNA, pulsed field gel electrophoresis, [7], [8], [9], [10], [11], [12], [13]. A major drawback of such DNA typing methods is a lack of comparability despite a high resolution power, whereas auxotyping, serotyping and plasmid typing methods, individually, have a much lower resolution power [14], [6]. More recent technological developments enabling high-throughput, low-cost nucleotide sequencing, have had a significant impact on the application of DNA-sequencing typing methods for *N. gonorrhoeae* [15]. Two of these methods, *Neisseria gonorrhoeae* multiangen sequence typing (NG-MAST) and *porB* DNA sequence analysis, target two hypervariable genes encoding outer surface proteins [16], [17], [15]. Another DNA-sequencing based typing method, multilocus sequencing typing (MLST), uses internal fragments of 7 housekeeping genes [18].

To evaluate the impact of different genetic markers, on the genetic relatedness of gonococcal isolates and subsequently on genotyping analysis of this human pathogen, we analyzed 10 housekeeping genes [18], [19] which are subject to purifying selection (i.e. removal of alleles that are deleterious) and slow evolution and compared them to the hypervariable *porB* DNA sequence. Since these two groups of genetic markers, housekeeping and antigenic loci, have quite different genetic and evolutionary characteristics, we aimed to investigate their possible influence and outcome on genotyping analyses using the same panel of *N. gonorrhoeae* isolates collected worldwide. We examined 80 gonococcal isolates originating in China, North and South America that were collected over different time periods. In addition, we revealed a geographic population structure of tested *N. gonorrhoeae* isolates and their evolutionary relationship, using a combination of the concatenated sequence of the housekeeping loci and molecular evolutionary analyses.

## Materials and Methods

### *Neisseria gonorrhoeae* isolates

80 *N. gonorrhoeae* isolates from six geographic locations worldwide were analyzed. In 2008, 41 isolates were obtained from the Saskatchewan Disease Control Laboratory (SDCL) Canada, and 23 were collected at the STD Clinic of the Shanghai Skin Disease and STD Hospital, Shanghai, China. Another 14 *N. gonorrhoeae* isolates were collected between 1982 and 1986 in South America, (Argentina, Venezuela and Chile) and were part of our culture collection [20]. Two other *N. gonorrhoeae* isolates were American in origin and are well characterized laboratory isolates, F-62 and FA1090 [21]. Ethical approval was obtained from the Ethics Board of the University of Saskatchewan (Canada) and the Ethics Committee of the Shanghai Municipal Bureau of Public Health (China).

### *porB* analysis

*PorB* was typed as described previously and included an analysis of 85% of the entire gene [22], [17]. *PorB* was amplified with porB-F and porB-R primers [23], (Invitrogen, Burlington, ON, Canada) and PCR amplification was performed as previously described [17].

### Target loci of 10 housekeeping genes, primers and PCR amplification

Based on two previous studies, Bannett et al., [18] and Viscidi and Demma [24], which examined polymorphisms in various *N. gonorrhoeae* genes encoding enzymes for intermediary metabolism, we selected 10 housekeeping genes for analysis, including *abcZ*, *adk*, *aroE*, *glnA*, *gdh*, *pyrD*, *pdhC*, *gnd, fumC* and *pgm*. These loci were selected based on their polymorphisms to ensure an application of informative markers distributed throughout the gonococcal genome.

Novel primers were designed for each housekeeping gene using the Primer 3 program [25]. Existing gonococcal DNA sequences deposited in the GenBank database (http:/blast.ncbi.nlm.nih.gov/Blast.cgi) were used as templates to design oligonucleotide primer pairs (Table 1) which bracketed the most polymorphic regions of the selected housekeeping genes. The primers showed high specificity and reliability in amplifying this diverse isolate collection. Two well characterized laboratory strains F-62 and FA 1090 (21) were used as the standard strains for validation of the PCR protocol. Polymerase chain reaction amplifications were carried out in a 50 µL volume containing ∼15 ng of template DNA, 20 nM Tris-HCL (pH 8.4), 50 mM KCl, 2 mM MgCl_2_, 200 µM each of deoxynucleoside triphosphatese (dNTPs), 0.5 µM of each primer and 2 U of *Taq* polymerase (Invitrogene Life Technologies, Canada). Temperature conditions included an initial 95°C denaturation step for 4 min., followed by 30 cycles of: denaturation at 95°C for 60 sec., annealing at 55°C for 30 sec., amplification at 72°C for 30 sec. and a final extension step at 72°C for 10 min.

**Table 1:** Primer pairs used for PCR-s for 10 housekeeping genes of *N. gonorrhoeae*.

	Putative function	Length (bp)	Primername	Sequence (5′→3′)
*adk*	Adenylate kinase	520	adk-F adk-R	CGTTCGGCATTCCGCAAATCTCT CGACTTTGATGTATTTCGGCGC
*pgm*	Phosphoglucomutase	435	pgm-F pgm-R	GAACACGGCGGAGAAGCCATAATG CTTGCGTATCCGCTTCAAAACGCA
*aroE*	Shikimate dehydrogenase	695	aroE-F aroE-R	GATTCATCAGCAATTTGCCCTTCA CCGCGCCAGAGGGCGTAGGAAGC
*pdhC*	Pyruvate dehydrogenase	414	pdhC-F pdhC-R	GTTCCGGTACGATTCTGCAAGAAG CGGTTTCTTTGCTGACTTTGCCT
*fumC*	Fumarate hydratase class II	535	fumC-F fumC-R	GCCATCCCGAATACGCTGAAATAG CGATTTTGCGGTTTAACGCAGTAAC
*pyrD*	Dihydroorotate dehydrogenase	592	pyrD-F pyrD-R	GATGATGCCGTCCATTTCGACGGA TATAAAACTGATGGGTATGGATTTGC
*gnd*	6-Phosphogluconate dehydrogenase	549	gnd-F gnd-R	GAGAAAATCCTCGATACGGCAGGGCA GGTCGTATAGCCGTCCAAGAACGT
*glnA*	Glutamine synthetase	537	glnA-F glnA-R	GTTTGAAAACGGACCGGCGTTTGA TTCCATTTGGCTGCCGGTACCGAC
*gdh*	Glucose-6-phosphate 1 dehydrogenase	630	gdh-F gdh-R	ATGTTCGAGCCGCTGTGGAACAA CTTCAACGGCCTTGCCCAAATCC
*abcZ* a	ATP-binding protein	498	abcZ-F abcZ-R	AATCGTTTATGTACCGCAGG GAGAACGAGCCGGGATAGGA

a The *abcZ* primer pairs were developed by Bennett et al. (2007).

### DNA sequencing and data analysis

Amplicons were purified by a PCR purification kit (Qiagen, Mississauga, Ontario, Canada) and their nucleotide sequences on both strands were determined using an Applied Biosystems 3730×1 DNA Analyzer (Plant Biotechnology Institute, National Research Council of Canada, Saskatoon, Saskatchewan, Canada). DNA sequences were analyzed using DNASTAR Lasergene 7 (DNASTAR Inc., United States). All sequences for each locus were compared and every unique sequence was assigned as a distinct allele using Sequence Output (www.mlst.net). Each unique combination of allele numbers was assigned as a different sequence type (ST). The average frequencies of synonymous (dS) and nonsynonymous (dN) substitutions per site were calculated for each locus using the Nei-Gojobori method [26]. The analysis of average pairwise distance was conducted using the Maximum Composite Likelihood method with pairwise deletion option for gaps [27], [28]. The discriminatory power of single gene sequence and the concatenated DNA sequences was revealed using a single numerical index of discrimination [29]. MEGA4 [28] was used to construct phylogenetic trees from concatenated sequences of the housekeeping genes and individual *porB* sequences, respectively by using a combination of several methods including: unweighted pair group method with arithmetic mean (UPGMA) [30] neighbour joining [31] and minimum evolution methods [32] with p-distance as well as Kimura 2-parameter [33] substitution models. In order to predict the ancestral profiles and clonal complexes, each genotype identified in this study was analyzed by eBURST [34]. For identification of clonal complexes, the most stringent default group definition was used, where all STs have to be single locus variants of at least one ST in the clonal complex. The initial eBURST diagrams were created at http://eburst.mlst.net and edited as required to produce the final figure. Editing did not change any of the links between the STs and was done to improve the clarity of the diagram.

### Detection of recombination in housekeeping genes and porB gene sequences

*porB* DNA sequences and individual and concatenated sequences of the housekeeping genes were analyzed for putative recombination events using a new version of Recombination Detection Program (RPD3) [35]. To reveal recombination events in DNA sequences, a range of non-parametric recombination detection methods was used including RDP [36], MaxChi [37], Chimeara [38], 3SEQ [39], GENECONV [40], and SiScan [41]. Putative recombination events detected by these non-parametric recombination detection methods were confirmed by the Likelihood Assisted Recombination Detection [42] method. The highest acceptable P-value, the highest acceptable probability that sequences could share high identities in potentially recombinant regions due to chance alone, was set to 0.05 with Bonferroni correction.

### Nucleotide sequences and accession numbers

Novel nucleotide sequences were deposited in GenBank. Accession numbers for the DNA sequences of the different housekeeping genes extended from HM359017 to HM359073 and the accession numbers for the *porB* sequences ranged from HM481249–481254.

## Results

### Housekeeping genes

Sequencing 10 housekeeping gene fragments in each of 80 gonococcal isolates revealed the existence of 52 polymorphic sites (Table 2), ranging from 2 for *pgm*, *pdhC*, and *adk* loci to 17 for the *glnA* locus. The high polymorphism of the *glnA* locus owned to haplotype (allele) 7, where a cluster of 13 polymorphic sites was found over a short 55- bp region of the locus. Except for *glnA* where all variable sites were concentrated in a ∼130 bp gene segment, the polymorphic sites for all other loci were randomly distributed across the entire locus. The mean of the pairwise divergence was 0.002 with the most divergent locus being *glnA* (0.0037) followed by *gnd* (0.0033) (Table 2).

**Table 2:** Genetic characteristics of gonococcal loci.

Locus	Location on chromosome (bp)^a^	No. of haplotypes	No. of polymorphic sites	Average pairwase distance	dN/dS ratio
*pgm*	368 712	3	2	0.0010	0.2720
*pdhC*	546 184	3	2	0.0014	0.2356
*gdh*	715 214	5	3	0.0014	0.4586
*gnd*	1 891 592	12	9	0.0033	0.4439
*glnA*	1 572 073	7	17	0.0037	0.0261
*pyrD*	1 723 162	7	5	0.0023	0.1381
*fumC*	992 677	7	5	0.0029	0.0867
*adk*	392 506	3	2	0.0003	0.6338
*aroE*	1 573 802	5	3	0.0013	0.2578
*abcZ*	853 097	6	4	0.0026	0.5599

a From the *N. gonorrhoeae* strain FA 1090 (http://www.ncbi.nlm.nih.gov).

Overall, 58 haplotypes were identified among the 80 isolates with the 10 loci (Table 2). The majority of the haplotypes were repetitive in the examined gonococcal population, whereas 14 haplotypes were observed only once. The *pyrD* locus showed the highest proportion of unique haplotypes (42.8%), followed by *abcZ* (33.3%) and *glnA* (28.5%). 26 haplotypes were uniquely distributed among specific geographic locations. The most numerous geographically distinct haplotypes, using the collection of 80 *N. gonorrhoeae* isolates, were found in Canada (n = 11), followed by China (n = 9), Venezuela (n = 2), the USA (n = 2), Chile (n = 1) and Argentina (n = 1).

The ratios of nonsynonymous and synonymous substitutions (dN/dS) for examined loci ranged from 0.026 to 0.633 (Table 2), indicating that purifying selection is a dominant force in the evolution of these genes. Using all 10 loci, the 80 gonococcal isolates analyzed were resolved into 41 sequence types (STs) **See Table S1**. The index of discrimination (ID) for these 10 housekeeping loci was 0.953.

**Figure 1:**
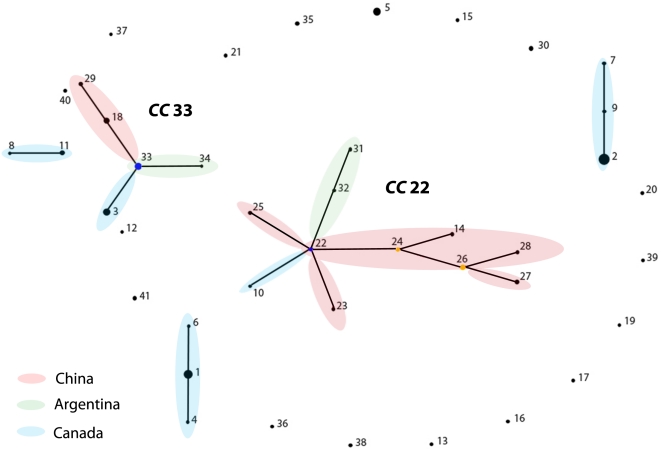
Population snapshot of 80 *N. gonorrhoeae* isolates from Canada, USA, Argentina, Venezuela, Chile and China as determined by eBURST analysis. Two major clonal complexes (CCs) were found, CC22 and CC33. CC22 consisted of eleven STs, with ST22 being the founder (blue circle) and two STs, ST24, and ST26, being subgroup founders (yellow circle). The CC33 consisted of five STs. Each circle represents a single ST and the size of each dot/circle is proportional to the number of isolates found. Evolutionary related STs were sharing nine out of ten alleles and they are connected by lines. The colored ellipses mark the STs of the same geographic origin.

Analysis by eBURST revealed the existence of two major clonal complexes (CC), CC22 and CC33 and three minor CCs (Fig. 1), among the population of geographically diverse *N. gonorrhoeae* isolates. ST22 was identified as a putative ancestral genotype of CC22, while ST24 and ST26 were identified as co-founders of the same clonal complex. Eight other STs - ST23, ST10, ST25, ST32, ST31, ST14, ST28, and ST27 - were found to descend from their ancestors ST22, ST24, and ST26. Another major CC, CC33, consisted of an ancestral genotype ST33, and four descent genotypes - ST3, ST34, ST18, and ST29. Minor CCs comprised two triplets (ST6-ST1-ST4; ST7-ST9-ST2) and one doublet (ST8-ST11; Fig. 1). Seventeen different STs were identified as singletons and could not be assigned to any existing CC (Fig. 1).

### *porB*

Based on their *porB* gene nucleotide sequences, *N. gonorrhoeae* isolates were divided into two groups, *porB1a* (PIA) (n = 13) and *porB1b* (PIB) (n = 67). The majority of tested isolates from South America (85.7%; n = 12) were associated with the PIA group while most isolates originating from China and North America (100%; n = 23 and 97.6%; n = 42, respectively), belonged to the PIB group. Comparative analysis of *porB* nucleotide sequences revealed a profound variability of the PIB, accounting for 72 polymorphic sites, which resulted in a 0.0314 average pairwase distance (Table 3). Overall, 40 polymorphic sites were detected in the PIA nucleotide sequences, resulting in a 0.0155 average pairwase distance (Table 3), which indicates a much lower variability compared to the PIB. The profound polymorphism of the PIB nucleotide sequences was partially associated with 3-, 5-, and 6-nucleotide insertions as well as 3-, and 5-nucleotide deletions identified in numerous *porB1b* isolates. The 13 PIA isolates shared 6 haplotypes, while the 67 PIB isolates included 48 haplotypes (Table 3). The *d*_N_/*d*_S_ ratio for *porB* was 1 (Table 3), much higher than observed for the housekeeping loci. This indicates the presence of selective pressure in *porB*. The ID for porB sequence analysis with these 80 isolates was 0.969.

**Table 3:** Genetic characteristics of *porB* nucleotide sequences for gonococcal isolates.

Locus	Length (bp)	No of isolates	Haplotypes	Polymorphic sites	Average pairwase distance	dN/dS ratio
*porB1a*	798	13	6	40	0.0155	1.0
*porB1b*	860	67	48	72	0.0314	1.0

### Phylogenetic analyses

**Figure 2:**
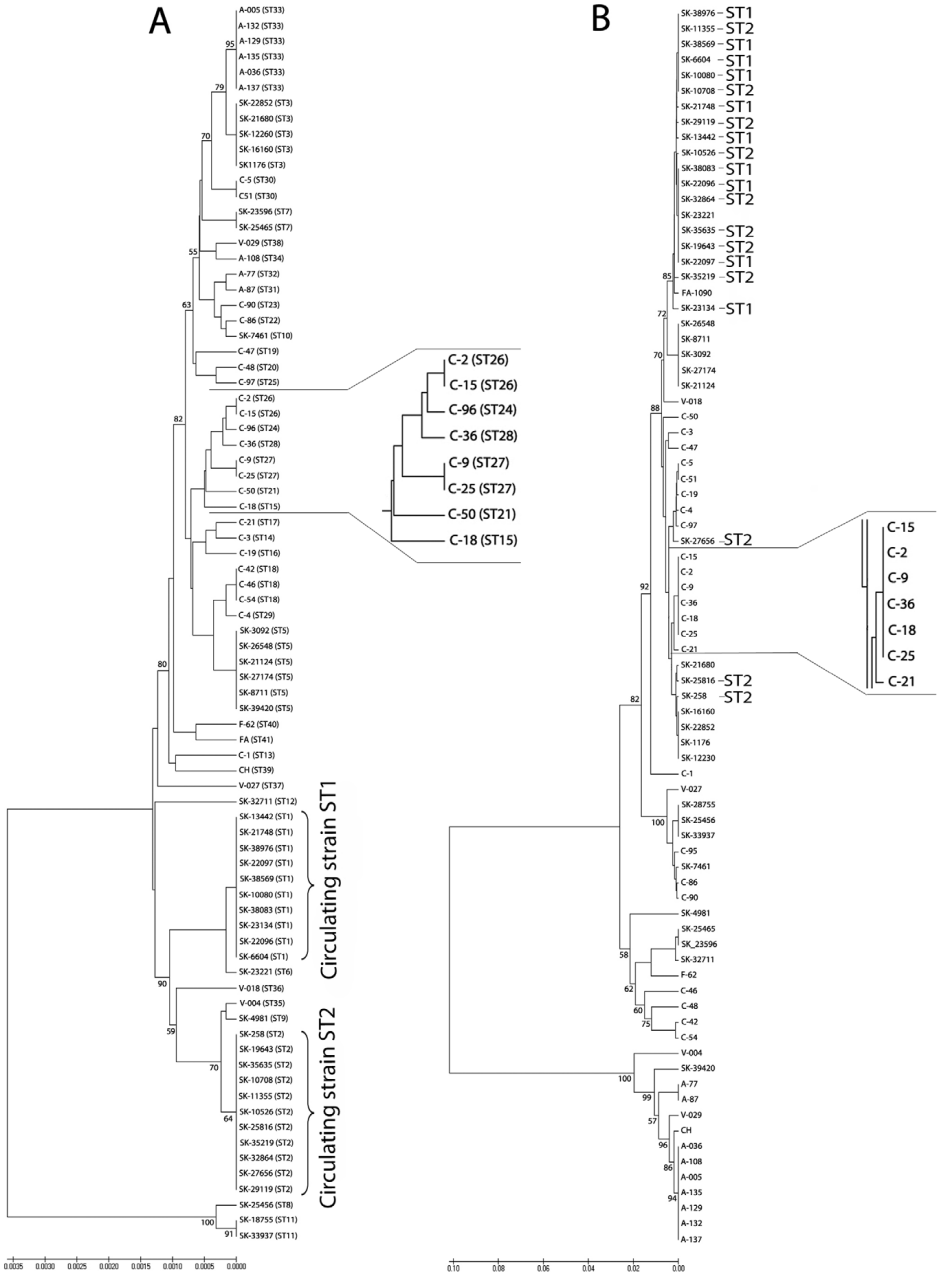
The UPGMA phylogenetic trees of A) the concatenated sequences of the housekeeping genes (*pyrD, abcZ, gnd, gdh, fumC, glnA, pgm, aroE, pdhC* and *adk*) and B) the *porB* sequences of *N. gonorrhoeae*. The tree was constructed using the matrix of pairwise differences. Bootstrap test was for 1000 repetitions. Each isolates of the MLST tree is marked by its sequence type (ST).The isolates of circulating strain ST1 and ST2, confined in northern part of the province Saskatchewan, are indicated by their STs in both the MLST and *porB* trees.

The trees obtained by the UPGMA, the minimum evolution, and the neighbor joining methods in combination with a couple substitution models were very similar with the same isolates being clustered together but being slightly different distributed along the trees. Thus, two UPGMA trees derived from the concatenated housekeeping genes and individual *porB* sequences (Fig. 2), respectively were analyzed and compared. The most notable difference between the housekeeping loci and the *porB* trees is that two major housekeeping gene clusters, ST1 and ST2, comprising 20 North American isolates, placed at the bottom of the tree (Fig. 2) while these isolates were mixed up in the *porB* tree forming several clades scattered throughout the *porB* tree (Fig. 2). Isolates from South America, originating from Argentina and Venezuela, were clustered together in both, the MLST and *porB* tree, indicating in these cases, the presence of the same phylogenetic signal in two different categories of genetic markers. Another important difference between these two groups of genetic markers was that the most numerous Asian cluster in the *porB* tree (Fig. 2), which comprised six isolates (C-15, C-2, C-9, C-36, C-18, and C-25), was resolved into a monophyletic cluster consisting of 5 lineages in the MLST tree (Fig. 2). Besides these two major differences, the remaining *N. gonorrhoeae* isolates were clustered very similarly in the MLST and *porB* trees.

### Assessment of recombination events

To determine how recombination events affected both *porB* DNA sequence, encoded by *porB* antigen gene, and DNA sequences encoded by housekeeping genes, several recombination detection analyses were performed. No recombination event was detected in any of the 10 housekeeping genes. In contrast, a single recombination event was detected in the PIA sequences, with breakpoint starting at 193 bp and ending at 298 bp position of aligned DNA sequences. Nine recombinant PIA gonococcal isolates from South America were noted A-005, A-036, A-108, A-129, A-132, A-137, A-135, V-029, and CH 811, while isolate A-77 was detected (99.6%) as a donor. Methods, number of individual recombination events and their P-values are shown in Table 4. No recombination was found in three PIA isolates: SK-39420, A-87, and V-004. The majority (94%; n = 63) of PIB isolates were recombinants, whereas four isolates, SK-4981, SK-25465, SK-23596, and SK-32711, all from Canada, had no detected recombination. The most numerous recombination event (Table 4), beginning at 27 bp and ending at 493 bp positions of the aligned DNA sequences, was identified in isolates from North America, China, and South America. Another recombination, starting at 602 bp and ending at 718 bp positions, was found (p<0.002) among 8 isolates from North America and China. Three recombination events were minor; a DNA fragment of 279 bp was found to be recombined (p<0.0000001) in three isolates from China (C-48, C-42, and C-54) and fragments of 92 bp and 73 bp, respectively, were recombined (p<0.05) in C-42 and C-54.

**Table 4:** Two major recombination events at PIA and PIB nucleotide sequences.

	*PorB1a*		*porB1b*	
Methods	No. of recombinants	P-value	No. of recombinants	P-value
RDP	8	0.0017	47	0.0009
GENECONV	7	0.0360	50	0.005
Max Chi	9	0.0089	50	0.00002
Chimaera	7	0.0064	47	0.00009
Si Scan	9	0.0005	50	4.319×10^−11^
3 Seq	9	0.0032	50	0.001

## Discussion

Effective strain typing plays a pivotal role in the surveillance of *N. gonorrhoeae* isolates, providing accurate information for the identification of predominant strains, which, in turn, can be used to improve control measures and implement successful interventions. A typing method used for surveillance should be highly accurate and reliable so that epidemiologically unrelated individuals can be separated from each other by genetic fingerprinting and at the same time that highly predominant strains which circulate through population can be accurately detected by the same typing method [15].

In this study, using a sequence-based approach of two different types of genetic marker, housekeeping genes and an antigen encoding gene, of 80 geographically and temporally diverse isolates of *N. gonorrhoeae* isolates, we empirically validated the impact of these markers on genotyping of gonococcal isolates. Although there was a degree of consistency between the concatenated sequences of the housekeeping loci and *porB* locus, a most notable difference observed between the respective phylogenetic trees was a discordant topology observed in North American isolates. Strains that formed two major North American clusters in the housekeeping tree were mixed up and scattered across the *porB* tree (Fig. 2). Using data from the Saskatchewan Disease Control Laboratory, we found that all isolates forming clusters “ST1” and “ST2” were restricted to the northern part of the province, suggesting that ST1 and ST2 isolates represent distinct *N. gonorrhoeae* strains circulating in a confined geographic region. In addition, our recent study (S. Vidovic and J. R. Dillon, unpublished data) showed that these two STs represent two predominant strains, circulating within the province of Saskatchewan over a 4-year period (2005–2008). A possible explanation for the failure of the *porB* typing method to detect and separate these two circulating strains of *N. gonorrhoeae* is that some regions of the *porB* gene are under strong positive immune selection as shown by the *d_N_*/*d*_S_ ratio and recombination analysis. This would impose a distorting effect on the genetic relatedness of the isolates. Using *Borrelia burgdorferi* as a model organism, Margos et al., [43] demonstrated that the concatenated sequences of housekeeping genes were capable of capturing geographic population structure of the organism. In contrast, the same collection of *B. burgdorferi* isolates analyzed by *ospC*, a hypervariable gene encoding the immunodominant outer surface protein C [44], showed the lack of ability to capture the geographic structure of a *B. burgdorferi* population. These results provide important evidence that the choice of genetic marker has a profound effect on the interpretation of epidemiological or population genetics results. Concatenated sequences of housekeeping genes can provide clonal stability further allowing discrimination of circulating strains or different bacterial ecotypes. In contrast, application of the commonly used genetic markers which are based on hypervariable DNA regions may result in an absence of phylogenetic signal indicating the presence of circulating strains or distinct bacterial ecotypes.

Another important difference between the concatenated sequences of the housekeeping genes and the *porB* was that the most numerous Asian cluster observed in the *porB* tree (Fig. 2). which comprised six isolates (C-15, C-2, C-9, C-36, C-18, and C-25), was resolved into a monophyletic cluster consisting of 5 lineages in the housekeeping tree (Fig. 2). In addition, our recent study using NG-MAST typing (M. Liao and J. R. Dillon, unpublished data) showed that the same group of *N. gonorrhoeae* isolates was clustered which further points that the concatenated sequences of the housekeeping genes can achieve a high discriminatory power. Although Simpson's index of diversity was slightly higher for the *porB* analysis, this index does not take the distorting effect of recombination into account. For instance, using the concatenated sequences of the housekeeping genes, 11 epidemiologically linked *N. gonorrhoeae* isolates were clustered together, representing one strain, ST2. In contrast, using *porB* genetic marker only 3 of these isolates were clustered together, while the other isolates were dispersed across the tree, generating additional 7 lineages. This example shows that recombination events occurring in *porB* drifts otherwise epidemiologically linked *N. gonorrhoeae* isolates from each other, generating more lineages.

The low *d_N_*/*d*_S_ ratios and the absence of recombination events for all ten housekeeping genes indicates that these genes are most likely not subjected to any selective pressure and they potentially retain signatures of longer-term evolutionary relationships. The evolutionary relationships between gonococcal isolates obtained from six worldwide locations, we performed eBURST analysis using all ten housekeeping genes identified 2 major clonal complexes, C22 and CC33. The founder of CC22 was, ST22, identified in China with the main direction of descent being established via ST24, first subfounder, and ST26, the second subfounder, which also originated in China. Additional four descendant genotypes were identified with two, ST25 and ST23, from China, one, ST10 from Canada and one, ST32, from Argentina. The ancestral genotype of another clonal complex CC33 was ST33, the circulating strain found in Argentina. CC33 possesses three descendent genotypes, ST34 (Argentina), ST3 (Canada), and ST18 (China). The eBURST analysis indicates that the smaller portion of the gonococcal population from Canada likely originated from somewhere else (South America and Asia). All minor clonal complexes were found in Canada, which most likely suggest that they represent an endemic gonococcal population. These endemic genotypes have been segregated for long enough to accumulate their own mutations and are diversifying and spreading over this geographic area.

The present study suggests that the choice of genetic marker, housekeeping or antigen genes, used for typing *N. gonorrhoeae* isolates will most likely have a profound effect on interpretation of genotyping results. Our study showed that the predominant *N. gonorrhoeae* strains from Saskatchewan, Canada, isolated in 2008 can be detected using the concatenated sequences of the housekeeping genes but not using a hypervariable genetic marker, *porB*. The concatenated sequences of the housekeeping genes retain the phylogenetic signals of the circulating strains and successfully identify them over a long period of time. On the other hand, the hypervaribale *porB* DNA sequence can be subjected to frequent mutation and recombination and therefore this type of genetic marker tends to introduce a distorting effect among genetically related isolates which may lead to a failure to detect the predominant strains of *N. gonorrhoeae* that circulate within a community over an extended period of time. Taken together, our data suggests that most likely a typing method based on antigen gene marker can be applied successfully only during most recent outbreaks. Application of these genetic markers in any other situation may result in more or less skewed picture. In addition, the concatenated sequences of the housekeeping genes can provide a high resolution power, discriminating unrelated strains. Using eBURST analysis, the study reveals that the gonococcal population from Canada was a mixture of endemic and introduced genotypes, with the endemic genotypes being predominant.

## Supporting Information

Table S1 Details of *N. gonorrheae* isolates used in this study.
(DOC)
