# Supplementary material for: Influence of Conserved and Hypervariable Genetic Markers on Genotyping Circulating Strains of Neisseria gonorrhoeae
Source: PLoS One. 2011 Dec 7;6(12):e28259. doi: 10.1371/journal.pone.0028259 (PMC3233552; doi:10.1371/journal.pone.0028259)
Supplement: Table S1 — Details of N. gonorrheae isolates used in this study. (DOC) [file pone.0028259.s001.doc]

**Table S1.** Details of *N. gonorrheae* isolates used in this study.

|  |  |  |  |  | Patients |  |
| --- | --- | --- | --- | --- | --- | --- |
| Isolate | ST | Year | Geographic origin | Gender | Age | Site of infection |
| SK-21748 | 1 | 2008 | Canada | Female | 19 | Vagina |
| SK-25816 | 2 | 2008 | Canada | Male | 23 | Penis |
| SK-27656 | 2 | 2008 | Canada | Male | 22 | Urethra |
| SK-29119 | 2 | 2008 | Canada | Male | 34 | Urethra |
| SK-22097 | 1 | 2008 | Canada | Female | 16 | Vagina |
| SK-22096 | 1 | 2008 | Canada | Female | 16 | Cervix |
| SK-19643 | 2 | 2008 | Canada | Male | 19 | Penis |
| SK-16160 | 3 | 2008 | Canada | Male | 48 | Urethra |
| SK-13442 | 4 | 2008 | Canada | Female | 19 | Cervix |
| SK-12230 | 3 | 2008 | Canada | Female | 17 | Cervix |
| SK-10708 | 2 | 2008 | Canada | Male | 20 | Urethra |
| SK-10526 | 2 | 2008 | Canada | Female | 17 | Vagina |
| SK-8711 | 5 | 2008 | Canada | Male | 19 | Urethra |
| SK-6604 | 1 | 2008 | Canada | Male | 22 | Urethra |
| SK-3092 | 5 | 2008 | Canada | Male | 21 | Urethra |
| SK-258 | 2 | 2008 | Canada | Male | 20 | Urethra |
| SK-22852 | 3 | 2008 | Canada | Female | 20 | Cervix |
| SK-23221 | 6 | 2008 | Canada | Male | 23 | Urethra |
| SK-23596 | 7 | 2008 | Canada | Male | 29 | Urethra |
| SK-39420 | 5 | 2008 | Canada | Male | 22 | Eye |
| SK-38569 | 1 | 2008 | Canada | Female | 35 | Cervix |
| SK-38083 | 1 | 2008 | Canada | Female | 20 | Urethra |
| SK-27174 | 5 | 2008 | Canada | Male | 63 | Urethra |
| SK-26548 | 5 | 2008 | Canada | Female | 20 | Cervix |
| SK-25456 | 8 | 2008 | Canada | Male | 70 | Urethra |
| SK-25465 | 7 | 2008 | Canada | Male | 47 | Urethra |
| SK-23134 | 1 | 2008 | Canada | Female | 18 | Cervix |
| SK-1176 | 3 | 2008 | Canada | Female | 34 | Cervix |
| SK-21124 | 5 | 2008 | Canada | Female | 28 | Cervix |
| SK-21680 | 3 | 2008 | Canada | Female | 29 | Cervix |
| SK-32864 | 2 | 2008 | Canada | Female | 19 | Vagina |
| SK-35635 | 2 | 2008 | Canada | Female | 18 | Cervix |
| SK-38976 | 1 | 2008 | Canada | Male | 46 | Penis |
| SK-10080 | 1 | 2008 | Canada | Female | 26 | Vagina |
| SK-4981 | 9 | 2008 | Canada | Male | 49 | Urethra |
| SK-7461 | 10 | 2008 | Canada | Female | 31 | Cervix |
| SK-11355 | 2 | 2008 | Canada | Male | 48 | Penis |
| SK-35219 | 2 | 2008 | Canada | Male | 26 | Urethra |
| SK-28755 | 11 | 2008 | Canada | Female | 35 | Vagina |
| SK-33937 | 11 | 2008 | Canada | Female | 23 | Cervix |
| SK-32711 | 12 | 2008 | Canada | Male | 21 | Urethra |
| C-1 | 13 | 2008 | China | Male | 73 | Urethra |
| C-3 | 14 | 2008 | China | Male | 45 | Urethra |
| C-18 | 15 | 2008 | China | Male | 49 | Urethra |
| C-19 | 16 | 2008 | China | Male |  | Urethra |
| C-21 | 17 | 2008 | China | Male | 34 | Urethra |
| C-42 | 18 | 2008 | China | Male | 25 | Urethra |

**Table S1.** - Continued

|  |  |  |  |  | Patients |  |
| --- | --- | --- | --- | --- | --- | --- |
| Isolate | ST | Year | Geographic origin | Gender | Age | Site of infection |
| C-47 | 19 | 2008 | China | Male | 39 | Urethra |
| C-46 | 18 | 2008 | China | Male | 58 | Urethra |
| C-48 | 20 | 2008 | China | Male | 22 | Urethra |
| C-50 | 21 | 2008 | China | Male | 45 | Urethra |
| C-54 | 18 | 2008 | China | Male | 35 | Urethra |
| C-86 | 22 | 2008 | China | Male | 27 | Urethra |
| C-90 | 23 | 2008 | China | Male | 28 | Urethra |
| C-95 | 24 | 2008 | China | Male | 38 | Urethra |
| C-97 | 25 | 2008 | China | Male | 42 | Urethra |
| C-2 | 26 | 2008 | China | Male | 39 | Urethra |
| C-9 | 27 | 2008 | China | Male | 41 | Urethra |
| C-15 | 26 | 2008 | China | Male | 34 | Urethra |
| C-25 | 27 | 2008 | China | Male | 27 | Urethra |
| C-36 | 28 | 2008 | China | Male | 43 | Urethra |
| C-4 | 29 | 2008 | China | Male | 31 | Urethra |
| C-5 | 30 | 2008 | China | Male | 24 | Urethra |
| C-51 | 30 | 2008 | China | Male | 52 | Urethra |
| A-87 | 31 | 1986 | Argentina |  |  |  |
| A-77 | 32 | 1984 | Argentina |  |  |  |
| A-137 | 33 | 1984 | Argentina |  |  |  |
| A-036 | 33 | 1984 | Argentina |  |  |  |
| A-005 | 33 | 1986 | Argentina |  |  |  |
| A-132 | 33 | 1986 | Argentina |  |  |  |
| A-135 | 33 | 1985 | Argentina |  |  |  |
| A-129 | 33 | 1984 | Argentina |  |  |  |
| A-108 | 34 | 1984 | Argentina |  |  |  |
| V-004 | 35 | 1985 | Venezuela |  |  |  |
| V-018 | 36 | 1985 | Venezuela |  |  |  |
| V-027 | 37 | 1985 | Venezuela |  |  |  |
| V-029 | 38 | 1985 | Venezuela |  |  |  |
| CH 811 | 39 | 1982 | Chile |  |  |  |
| F-62 | 40 | 1960’ | USA |  |  |  |
| FA 1090 | 41 | 1960’ | USA |  |  |  |
